# Supplementary material for: MiRNAs in Lung Adenocarcinoma: Role, Diagnosis, Prognosis, and Therapy
Source: Int J Mol Sci. 2023 Aug 27;24(17):13302. doi: 10.3390/ijms241713302 (PMC10487838; doi:10.3390/ijms241713302)
Supplement: Supplementary file 1 [file ijms-24-13302-s001.zip › appendix S1.pdf]

Tumor suppressor miRNAs in LUAD

| MiRNA | Possible Targets | References (PMID) |
|---|---|---|
| miR-1 | FAM83A | 33266425 |
| miR-7 | BCL-2 | 21750649 |
| miR-15a/miR-16 | cyclin D1 | 19549910 |
| miR-16 | TFAP2A | 33824285 |
| miR-22 | ErbB3 | 22484852 |
| miR-23b | cyclin D1 | 28976503 |
| miR-24 | SOX18 | 29115529 |
| miR-26a | HMGA1 | 28000891 |
| miR-26b | COX-2 | 26744864 |
| miR-27a | MAP17 | 33280497 |
| miR-27b | Snail1 | 31887300 |
| miR-29a | CEACAM6 | 25171863 |
| miR-29b | MMP2 | 26063204 |
| miR-29c | VEGFA | 28241836 |
| miR-30a | CNPY2 | 31894275 |
| miR-30b/c | Rab18 | 25249344 |
| miR-32 | SMAD3 | 33758603 |
| miR-33a | CAND1 | 28871425 |
| miR-33b | ZEB1 | 26459797 |
| miR-34 | Cdh2 and Fn1 | 30700696 |
| miR-92a | Sprouty 4 | 35484993 |
| miR-92b | EZH2 | 32271434 |
| miR-98 | TGFBR1 | 30387848 |
| miR-99a | FAM64A | 32932948 |
| miR-99b | FZD8 | 31040702 |
| miR-100 | HOXA1 | 32364673 |
| miR-101 | EG5 | 30049386 |
| miR-107 | STK33 | 32395291 |
| miR-122 | Forkhead box O | 29434994 |
| miR-124 | AKT2 | 32873299 |
| miR-125a | STAT3 | 31930562 |
| miR-125b |  | 35187068 |
| miR-126 | EZH2 | 35322532 |
| miR-129 | HMGB1 | 31186804 |
| miR-130 | EZH2 | 31773700 |
| miR-132 | ZEB2 | 32500672 |
| miR-133 | ELOT2 | 28503944 |
| miR-135a | RAB1B | 32710726 |
| miR-136 | Smad2/Smad3 | 25198664 |

| | | |
|---|---|---|
| miR-138 | ZEB2 | 30712885 |
| miR-139 | CCNB1 | 35181869 |
| miR-140 | TYMS | 34818974 |
| miR-142 | NR2F6 | 31168689 |
| miR-143 | MCM4 | 31514295 |
| miR-144 | EZH2 | 30280514 |
| miR-145 | EGFR/NUDT1 | 21289483 |
| miR-147 | BDNF | 32724437 |
| miR-148a | MAP3K9 | 35388129 |
| miR-148b | ALCAM | 31883226 |
| miR-149 | RAP1B | 32432747 |
| miR-150 | TNS4 | 31052206 |
| miR-152 | TNS1 | 33269380 |
| miR-153 | Jagged1 | 32375892 |
| miR-154 | BMI-1 | 29928380 |
| miR-155 | Smad2 | 30013636 |
| miR-181a | ETS1/STK16 | 35092121 |
| miR-181b | Sox6 | 30580904 |
| miR-181d | CDKN3 | 30628487 |
| miR-182 | CTTN | 21503569 |
| miR-184 | C1QTNF6 | 35578071 |
| miR-185 | KLF7 | 29716672 |
| miR-186 | cyclinD1,CDK2,CDK6 | 23204228 |
| miR-187 | FGF9 | 31884893 |
| miR-188 | SIX1 | 32016974 |
| miR-192 | CCNB1 | 33425490 |
| miR-193a | Slug | 30685413 |
| miR-193b | CCND1 | 31262974 |
| miR-194 | BMP1, p27 | 23584484 |
| miR-195 | PTBP1 | 35600383 |
| miR-198 | Livin | 28765921 |
| miR-199a | AGR2 | 34632938 |
| miR-199b | K-Ras | 30987652 |
| miR-200 | Flt1 | 21115742, 30208739 |
| miR-202 | RRM2 | 36660663 |
| miR-203 | ZEB2 | 27733346 |
| miR-204 | PCNA-1 | 30628638 |
| miR-206 | MET | 27446414 |
| miR-215 | | 28454402 |
| miR-216a | Bcl-2 | 30425570 |
| miR-216b | PBK | 29805502 |
| miR-217 | SIRT1 | 32267139 |
| miR-218 | ERO1A | 35441565 |

| | | |
|---|---|---|
| miR-222 | | 34240140 |
| miR-223 | NLRP3 | 36276078 |
| miR-296 | PRKCA | 28751441 |
| miR-320a | STAT3 | 28106481 |
| miR-320b | HNF4G, IGF2BP2 | 33758932 |
| miR-325 | HMGB1 | 25776482 |
| miR-326 | PD-L1, B7-H3 | 34131111 |
| miR-328 | PYCR1 | 33706104 |
| miR-330 1270 | Pol I | 36253542 |
| miR-331 | MLLT10 | 32765078 |
| miR-335 | CCNB2 | 32636645 |
| miR-338 | AKAP12 | 33747281 |
| miR-339 | BCL6 | 30333862 |
| miR-340 | p27 | 25151966 |
| miR-342 | E2F1 | 26483346 |
| miR-345 | RhoA | 34748526 |
| miR-363 | NEDD9, SOX4 | 31332786 |
| miR-370 | HMGA2 | 32719345 |
| miR-373 | BRF2 | 29025258 |
| miR-374a | TGFA | 27207663 |
| miR-374b | ITGB1 | 32364676 |
| miR-376a | c-Myc | 28741879 |
| miR-376b | KLF15 | 33015790 |
| miR-376c | LRH-1 | 27049310 |
| miR-377 | AKT1 | 30485528 |
| miR-379 | ARRB1 | 33173959 |
| miR-381 | ID1 | 22592211 |
| miR-382 | SAE1 | 35720620 |
| miR-383 | CIP2A | 28927114 |
| miR-384 | AEG-1 | 28938524 |
| miR-409 | SPIN1 | 30290307 |
| miR-422a | MMP2, MMP9 | 33300080 |
| miR-431 | DDX5 | 30720177 |
| miR-433 | Smad2 | 31445716 |
| miR-448 | IRS2 | 30912183 |
| miR-449a | KDM3A | 34044859 |
| miR-449b | | 32471413 |
| miR-450 | IRF2 | 27246609 |
| miR-451 | PSMB8 | 25150396 |
| miR-454 | TGFB2 | 33760169 |
| miR-455 | HOXB5 | 29170127 |
| miR-485 | Flot2 | 27262438 |
| miR-486 | SAPCD2 | 35467005 |

| | | |
|---|---|---|
| miR-488 | PYCR1 | 30605882 |
| miR-489 | USP48 | 35413838 |
| miR-490 | Wnt/β-catenin | 33154676 |
| miR-491 | FGF5 | 35866594 |
| miR-493 | RASL11B | 34296954 |
| miR-494 | IGF2 | 22151897 |
| miR-495 | HMGA2 | 30569167 |
| miR-496 | eIF3h | 34038061 |
| miR-497 | SMURF2 | 31581360 |
| miR-498 | HMGA2 | 29630114 |
| miR-503 | PI3K,p85,IKK-β | 24550137 |
| miR-504 | LOXL2 | 29156517 |
| miR-506 | TULP3 | 34874810 |
| miR-507 | ZEB2 | 31799665 |
| miR-508 | S100A16 | 36967030 |
| miR-509 | PLK1 | 27498003 |
| miR-511 | TRIB2 | 23071539 |
| miR-512 | IKBKBAS | 34702815 |
| miR-513b | HMGB3 | 30623409 |
| miR-515 | EEF2 | 32943920 |
| miR-516a | HIST3H2A | 30966836 |
| miR-516b | CDK2, MMP-2, MMP-9 | 28514208 |
| miR-519a | STAT3 | 32224524 |
| miR-519c | HIF-1α | 20233879 |
| miR-519d | VEGFA | 33116606 |
| miR-520c | AKT1, AKT2 | 30942957 |
| miR-532 | KRAS, MKL2 | 28474808 |
| miR-542 | cAMP | 28927388 |
| miR-545 | cyclinD1,CDK4 | 24505359 |
| miR-561 | P-REX2a | 31711559 |
| miR-563 | LIN28B | 31766078 |
| miR-564 | ZIC3 | 26498524 |
| miR-567 | CDK8 | 33911969 |
| miR-569 | c-FOS,HMGA2 | 29541173 |
| miR-576 | SGK1 | 30257988 |
| miR-577 | | 30628697 |
| miR-579 | CRABP2 | 35966249 |
| miR-582 | NOTCH1 | 31170211 |
| miR-584 | MMP-14 | 30628644 |
| miR-585 | hSMG-1 | 27743168 |
| miR-589 | HDAC5 | 28440397 |
| miR-593 | ICAM-1 | 32373966 |
| miR-598 | THBS2 | 36609437 |

| | | |
|---|---|---|
| miR-600 | METTL3 | 30774445 |
| miR-608 | AKT2 | 29075783 |
| miR-613 | CDK4 | 26744345 |
| miR-615 | IGF2 | 29562959 |
| miR-625 | Resistin | 32581589 |
| miR-628 | ING1 | 30372865 |
| miR-630 | CDC7 | 25255219 |
| miR-631 | E2F2 | 35353027 |
| miR-635 | YY1 | 27810784 |
| miR-637 | Akt1 | 34176781 |
| miR-638 | SOX2 | 24842609 |
| miR-641 | MDM2 | 28800790 |
| miR-647 | TRAF2 | 30349310 |
| miR-654 | PLK4 | 32884289 |
| miR-655 | PTTG1 | 31094297 |
| miR-660 | | 28124991 |
| miR-671 | MFAP3L | 34841435 |
| miR-675 | GPR55 | 25889562 |
| miR-708 | DNMT3A | 28972040 |
| miR-758 | HMGB | 30446524 |
| miR-760 | ROS1 | 29372517 |
| miR-767 | CLDN18 | 29169410 |
| miR-769 | HDGF | 34978893 |
| miR-770 | JMJD6 | 28882645 |
| miR-874 | MMP-2 | 23583374 |
| miR-876 | SPRR3 | 32273714 |
| miR-877 | ACP5 | 33222607 |
| miR-885 | IGF1R | 26554827 |
| miR-886 | TGF-β1 | 30230945 |
| miR-924 | RHBDD1 | 33041671 |
| miR-935 | E2F7 | 30203720 |
| miR-936 | E2F2 | 30210611 |
| miR-940 | FAM83F | 30280778 |
| miR-944 | STAT1 | 30881499 |
| miR-1179 | SPAG5 | 30180955 |
| miR-1205 | KRAS | 30906631 |
| miR-1225 | Sox9 | 33101465 |
| miR-1226 | FGF2 | 35164915 |
| miR-1227 | Spt16 | 32860308 |
| miR-1231 | | 34223779 |
| miR-1236 | KLF8 | 28842254 |
| miR-1247 | STAT5A | 35517418 |
| miR-1253 | WNT5A | 29415994 |

| | | |
|---|---|---|
| miR-1254 | HO-1 | 28749936 |
| miR-1256 | TCTN1 | 30008857 |
| miR-1258 | GRB2 | 30069987 |
| miR-1262 | ULK1, RAB3D | 28510306 |
| miR-1270 | Pol I | 36253542 |
| miR-1271 | mTor | 26692935 |
| miR-1284 | Myc | 28713980 |
| miR-1286 | PKM2 | 36817669 |
| miR-1296 | Wnt | 31897178 |
| miR-1297 | TRIB2 | 23071539 |
| miR-1298 | FAK, LAMB3 | 27698189 |
| miR-1299 | EGFR | 32801771 |
| miR-1304 | HO-1 | 27641735 |
| miR-1305 | MDM2 | 31807077 |
| miR-1321 | CDC20 | 35050556 |
| miR-1469 | stat5a | 26045996 |
| miR-1471 | FOXL1 | 33045131 |
| miR-1827 | MYC, FAM83F | 31595558 |
| miR-1908 | AKT1 | 27178817 |
| miR-1911 | mEAK-7 | 33364499 |
| miR-1915 | SET | 34774019 |
| miR-1976 | PLCE1 | 27063799 |
| miR-3666 | BPTF | 30481052 |
| miR-3941 | IGBP1 | 28012229 |
| miR-4429 | Wnt/β-catenin | 34491827 |
| miR-4732 | XPR1 | 35388387 |
