# Supplementary material for: MiRNAs in Lung Adenocarcinoma: Role, Diagnosis, Prognosis, and Therapy
Source: Int J Mol Sci. 2023 Aug 27;24(17):13302. doi: 10.3390/ijms241713302 (PMC10487838; doi:10.3390/ijms241713302)
Supplement: Supplementary file 1 [file ijms-24-13302-s001.zip › appendix S2.pdf]

Oncogenic miRNAs in LUAD

MiRNA	Possible Targets	References (PMID)
miR-9	ID4	34723712
miR-10b	KLF4	24216130
miR-15b	BCL2	32220063
miR-18a	IRF2	28471447
miR-19(a/b)	PTEN	26098000
miR-20a	PTEN	35857905
miR-20b	APC	31894264
miR-21	SET/TAF-Iα	31176779
miR-24	SOX7	29231262
miR-25	LATS2	31316723
miR-28	PTEN	32236614
miR-30e	PTPN13	28653805
miR-31	RAS/MAPK	26657862
miR-92a	NF2	30816526
miR-93	PTEN, RB1	29309884
miR-95	SNX1	24835695
miR-96	ARHGAP6	34338998
miR-103	KLF7	32582959
miR-106a	LKB1	34707704
miR-106b	PTEN	31954745
miR-122	N-cadherin, Vimentin	34884671
miR-127	EMT	27869168
miR-128	Drosha	29236960
miR-134	DAB2	31689617
miR-137	TFAP2C	28610956
miR-141	KLF9	31841191
miR-146a	TRAF6	33015045
miR-147b	MFAP4	31884109
miR-151a		32633369
miR-183	PECAM1	29749535
miR-196a	ANXA1, CCL2	33775710
miR-196b	RSPO2	33402849
miR-197	CYLD	29286108
miR-205	PTEN, PHLPP2	23856247
miR-208a	SRCIN1	31432113
miR-210	LOXL4	30633357
miR-212	Id3	32039486
miR-214	SUFU	29693173
miR-221	PTEN	34107168

miR-224	RASSF8	28770961
miR-297	GPC5	27554041
miR-301a	Runx3	31122259
miR-301b	DLC1	33754907
miR-323	TMEFF2	32009129
miR-346	XPC	27777383
miR-361	SMAD2	30365047
miR-362	Sema3A	30155491
miR-365	USP33	29743814
miR-367	FBXW7	28656290
miR-371b	SCAI	33103723
miR-375	ITPKB	26642205
miR-378	RBX1	29344280
miR-410	PTEN	29440630
miR-411	EMT	24833665
miR-421	FOXO1	33093915
miR-423	CADM1	33205911
miR-425	ADAM9	30038506
miR-429	PTEN, RASSF8	24866238
miR-483	RhoGDI1,ALCAM	24710410
miR-484	Apaf-1	28982084
miR-487a	OVOL2	29330288
miR-487b	MAGI2	24258346
miR-492		34818961
miR-499a	EMT	30978341
miR-500	ING1	30372865
miR-505	TP53AIP1	30864684
miR-510	SRCIN1	30982489
miR-514b	PI3K	36180981
miR-518b	FOXN1	32724361
miR-522	DENN/MADD	26783084
miR-543	MTA1	32410569
miR-550a	LIMD1	33194664
miR-552	Fibulin5	34736163
miR-572	KLF2	35587058
miR-574	mPGES-1	30922080
miR-587	CYLD	33378022
miR-590	OLFM4	28012926
miR-605	TNFAIP3	31452243
miR-616	SOX7	28765960
miR-619	RCAN1.4	32004570
miR-620	GPC5	24682381
miR-629	CELSR1	32108166

miR-645	TP53I11	32572880
miR-650	ING4	31611970
miR-657	SRCIN1	36033827
miR-661	RB1	28716024
miR-663	PUMA/BBC3,BTG2	29352138
miR-665	PTPRB	32269632
miR-744	c-Fos	34599436
miR-765	BMP6	34376998
miR-768	MMP-2, MMP-9	29048613
miR-802	menin	24994111
miR-873	SRCIN1	26807196
miR-875	SATB2	29196257
miR-937	INPP4B	27179609
miR-939	TIMP2	30272338
miR-942	FOXO1	34838826
miR-1181	AXIN1	34630639
miR-1197	HOXC11	31181445
miR-1204	PITX1	30549141
miR-1246	GSK-3β	30913872
miR-1269	tp53, caspase-9	29618932
miR-1275	Wnt/β-catenin	32194819
miR-1290	IRF2	29275213
miR-1293	PGM5	34616611
miR-1301	Thy-1	33692859
miR-1303		32508320
miR-1307	TRAF3	33061854
miR-1323	Cbl-b	32154175
miR-2355	ZCCHC14	34334103
miR-3607	APC	28866416
miR-3646	SORBS1	35196185
miR-3648	SOCS2	35037826
miR-4306	IGF2R	35070756
