# Supplementary material for: MiRNAs in Lung Adenocarcinoma: Role, Diagnosis, Prognosis, and Therapy
Source: Int J Mol Sci. 2023 Aug 27;24(17):13302. doi: 10.3390/ijms241713302 (PMC10487838; doi:10.3390/ijms241713302)
Supplement: Supplementary file 1 [file ijms-24-13302-s001.zip › appendix S3.pdf]

MiRNAs involved in ceRNA networks in LUAD

MiRNA	Possible Targets	References(PMID)
miR-7	IRS2	35844799
miR-9	CPEB3	33159015
miR-10a		32423237
miR-15a	B7H3	35920698
miR-17	QKI-5	30975029
miR-18b	VMA21	31297866
miR-20a	SLC7A5	35035814
miR-20b	CCND1	31686834
miR-22	BCL2	29030962
miR-23a		28059053
miR-25	FOXP2	32440153
miR-26a	E2F7	30257360
miR-27a	SFRP1	33282723
miR-27b	GSPT1	31190891
miR-29b	STAT3	30747209
miR-29c	MCL-1	35069696
miR-30a	PHTF2	31721438
miR-30b	ROCK1	35225646
miR-30c	SOX9	31541993
miR-32	SOX9, TWIST1	28800794
miR-33a	Twist1	31941509
miR-33b	GPAM	33821667
miR-34	PDL1	31949130
miR-93		29704631
miR-96	CYLD	31860169
miR-98	AKR1B10-ERK	30692511
miR-100	SMARCA5	35307327
miR-101	CISD1	35320929
miR-103	KLF4	31119790
miR-105	FHL1	31772674
miR-106a	MYLI9	34061466
miR-106b	FOXJ3	34724864
miR-107		30720176
miR-122	FSTL3	32280246
miR-124	SphK1	34738865
miR-125a	CSNK2A1	35266446
miR-125b		35244469
miR-126	COL1A1	35842617
miR-127	MED28	32196603

miR-128	GATA2	35538035
miR-129	CTNNB1	33753463
miR-130a	PDPK1	32820252
miR-133a	EMT	31005252
miR-133b	MMP9	29958139
miR-135a	SIRT1	30841451
miR-135b	TOX3	35661247
miR-136	MAT2A	34258296
miR-138	sirt6	31128573
miR-139	IGF1R	32734536
miR-140	E2F7	33569293
miR-141	KEAP1/NRF2	34136401
miR-142	CDK5	31363080
miR-143	HOXA10	33118120
miR-144	HOXA10	31381886
miR-145	MMP2	35441564
miR-146a/b	RHPN2	33877886
miR-147a	slug	30971296
miR-148a	ROCK1	34271873
miR-148b	PKM2	35924724
miR-149	FOXM1	31597523
miR-150	MMP14	31522616
miR-152		34455968
miR-153	S100A14	32922043
miR-154	SP3	36889558
miR-155	AXIN1	33506901
miR-181a	CARM1	35525959
miR-181b	KPNA4	33327962
miR-181c	ZIC2	34232917
miR-181d	ETS1	32099396
miR-182		31301086
miR-183	Hippo/YAP	35435136
miR-184	SF1	31897168
miR-185	MECP2	33937054
miR-186	NCAPG2	36059662
miR-188	KLF12	34644678
miR-193a	NETO2	34671432
miR-194	PD-L1	33777742
miR-195	GCNT3	35399076
miR-196b	LIN28B	29950144
miR-197	PTPN9	35156900
miR-198	KLF8	35134495
miR-199a	c-Myc	31488218

miR-199b	CAV1	31296840
miR-200	BiP	36802727
miR-202	EGFR	31788071
miR-203	SNAI	30617305
miR-204	ETS1	31889958
miR-205	c-Met	34616424
miR-206	NAMPT	33540574
miR-211	CDH4	32329852
miR-212	CBLL1	31646569
miR-215	SLC2A5	32903414
miR-216a	RAP2B	33023331
miR-217	FOXP1	29934982
miR-218	COL1A1	35842617
miR-219a	CAMK1D	33660800
miR-221	IRF2	34022918
miR-223	VEGFA	32052546
miR-224	PBX3	35342419
miR-296	KLF15	36774884
miR-298	c-Myc	33237626
miR-301a	SNX20	34604311
miR-301b	STARD13	34281560
miR-302c	FOXA1	30359741
miR-320a	LIMK1	32141553
miR-320b	BMI1	30592135
miR-320d	E2F1	33589572
miR-324	FBXO11, SP1	33061453
miR-326	BECN1	35012553
miR-328	SRCIN1	32323798
miR-330		35719962
miR-331	LAD1	33536022
miR-335	CTHRC1	35246517
miR-337	JAK2	31754348
miR-338	RAB14	32248836
miR-339	SLC7A11	35399708
miR-340	EDNRB	32774737
miR-342	DDX49	33089952
miR-346	APC	33968785
miR-361	SF3B3	30665425
miR-362	E2F1	31071530
miR-363	MDM2	31968395
miR-367	ZEB2	29339211
miR-370	HMGA2	32719345
miR-374b	SRSF7	32141554

miR-375	ACVRL1	35908770
miR-376a	EIF4G2	35196197
miR-376b	SULF1	32432744
miR-377	IGF1R	35668527
miR-378a	SOX7	32808728
miR-378c	SLC2A1	32960438
miR-379	IGF1	34592879
miR-381	CXCR4	31815619
miR-382	SPIN1	33061605
miR-383	VEGFA	35980157
miR-384	TRIM44	33442296
miR-409	SOD1	34801596
miR-410	HMGB1	32904350
miR-411	KPNA2	34903711
miR-421	TMEM100	35371319
miR-422a	KLK4	28177876
miR-423	MYBL2	31758653
miR-424	PLSCR4	31322545
miR-429	KCTD12	35426242
miR-431	ZEB1	34288814
miR-432	EMT	31281461
miR-433	MAPK8	33593390
miR-448	Bcl-2	29247949
miR-449a	TGIF2	31793741
miR-449b	NOTCH1	33191397
miR-450b	EZH2	29408583
miR-451	TRIM66	30481109
miR-455	UBE2V1	33311443
miR-485	WLS	33109341
miR-486	TEK	35946541
miR-488	ZNF281	33320427
miR-489	SOX4	31386568
miR-490	MAPK1	32494004
miR-491	ZNF703	32275336
miR-492		36117850
miR-493	ROCK1	34537072
miR-494	IGF1R	35668527
miR-495	FAM83A	33732373
miR-496	mTOR	29266795
miR-497	IGF1-R	30454699
miR-498	WWOX	32606933
miR-503	SEPT2	35046387
miR-506	HMGA2	32425695

miR-507	SOX4	34311177
miR-508	CRISP3	34504061
miR-509	SOX2, CD34	34715859
miR-510	PTEN	30840927
miR-511	PDL1	36947452
miR-512	p21	32858372
miR-513b	DUSP11	33116570
miR-514a 194	TWIST1	31645542
miR-515	ITGB8	33945401
miR-516a	FOXK1	35166053
miR-516b	SLC1A5	35482822
miR-519a	ZBTB5	31029744
miR-519b	RCCD1	31582214
miR-519d	HMGB1	32602212
miR-519e	S100A4	33173348
miR-522	Wnt	31190356
miR-524	NACC1	32735888
miR-525	VMA21	34507559
miR-526b	OSBPL5	35000595
miR-527	BRF2	30618167
miR-532	FOXR2	34494941
miR-539		32633366
miR-542	CCND1, CDK6	30275712
miR-543		33892399
miR-544a	FBXW7	29324317
miR-545	HS6ST2	35260044
miR-551b	FGFR1	34113122
miR-556	TEK	37005879
miR-558	MMP17, MMP1	33190405
miR-562	CYLD	33116647
miR-567	PTPRG	31012177
miR-573	E2F3	30309647
miR-577	STAT3	35475457
miR-578	HMGA2	34236145
miR-579	SOX4	34311177
miR-582	cyclin D1	35549985
miR-584	E2F5	32420810
miR-585	MMP16	34747300
miR-587	ODAM	34634673
miR-588	HOXA10	35872625
miR-593	CCND2	30722989
miR-598	Twist1	30240003
miR-600	HIF-1α	31241217

miR-605	MACC1	34493358
miR-607	ITGB1	34643030
miR-613	SphK1	33849377
miR-614	FAM83D	32228656
miR-615	AKT1	30556874
miR-622	HIF1-α	31668923
miR-623	MMP3	35076836
miR-625	CPSF7	31997940
miR-626	LIFR	33475233
miR-627	CCAR1	32945522
miR-629	TXNIP	35071487
miR-630	AEG-1	31749230
miR-634	GRB2	32160453
miR-635	PDL1	35723188
miR-636	PDL1	35167195
miR-638	FOSL2	33221767
miR-641	SETD7	33372601
miR-644a	PTBP1	33520363
miR-650	SLC34A2	31585300
miR-653	HK2	35379058
miR-655	Bcl-2	35473552
miR-656	xCT	36164726
miR-660	UBN2	36229758
miR-661	DOK7	34528912
miR-664a	PTBP1	33520363
miR-665	HEYL	34349799
miR-670	UHRF1BP1	33844396
miR-671	FZD4	36434577
miR-675	P53	31219199
miR-715	c-Myc	32706069
miR-744	MAFG	31211984
miR-758	MMP16	34747300
miR-760	SHCBP1	32003010
miR-761	TIMP2	31545237
miR-766	MAPK1	31939714
miR-767		32357143
miR-769	EGFR	35920610
miR-873	TUSC3	29790668
miR-874	STAT3	32699535
miR-875	SATB2	34080031
miR-876	WNT5A	33619796
miR-877	VEGFA	33869051
miR-885	Wnt10b	34872582

miR-890		34306585
miR-924	SRSF7	35037349
miR-940	BMPER	32922033
miR-942	TNS1	36581942
miR-944	ETS1	32099396
miR-1178	APITD1	35310916
miR-1179	SPAG5	31667785
miR-1180	AKT, GSK-3β	28744397
miR-1182	KLF8	32768951
miR-1183	PI3K/PKB	32236577
miR-1184	CCL22	35396377
miR-1197	MADD	33542659
miR-1202		35702784
miR-1206	BMPR2	35570745
miR-1207	EPB41L5	34285192
miR-1208	PI3K	33896835
miR-1224	ETV1	32308483
miR-1225	ErbbB2	33577030
miR-1228	TCF21	30176158
miR-1233	DUSP9	33348975
miR-1236	ZEB1	35342341
miR-1238	CLDN14	36098705
miR-1245	BRCA2	29891014
miR-1248	CCND2	33727831
miR-1249	HOXA13	35076814
miR-1252	FOXR2	33464477
miR-1253	RAB3D	32308420
miR-1256		30970529
miR-1258	AKT3	36593867
miR-1261	PIK3CA	35474604
miR-1267	ING3	35845928
miR-1270	PLAGL2	31911754
miR-1271	FRS2	32515146
miR-1275	LZTS3	33315502
miR-1276	HIF1α	32878637
miR-1278	WNT3A/5A	34093821
miR-1283	PDZD8	35415250
miR-1285	YTHDF1	34663343
miR-1286		31497197
miR-1287	PAK2	35333693
miR-1290	ACSL4	36482742
miR-1294	HMGA1	35114891
miR-1296	SIX1	35287543

miR-1298	GOT2	34077306
miR-1299		35441579
miR-1303		34386074
miR-1304	PPDPF	30954221
miR-1305	CyclinD1, MMP-2	35281529
miR-1322	SLC5A1	37007569
miR-1323	PDCD4	35460386
miR-1343	TGFBR1	32643848
miR-1827	SLC1A5	36192755
miR-1972	EGFR	33516270
miR-3163	SPP1	35603556
miR-3186	TNRC6B	31960988
miR-3196	SOX12	35275356
miR-3611	GCG	35071451
miR-3681	NEK2	34850664
miR-4677	ZEB1	31173403
