# Supplementary material for: MiRNAs in Lung Adenocarcinoma: Role, Diagnosis, Prognosis, and Therapy
Source: Int J Mol Sci. 2023 Aug 27;24(17):13302. doi: 10.3390/ijms241713302 (PMC10487838; doi:10.3390/ijms241713302)
Supplement: Supplementary file 1 [file ijms-24-13302-s001.zip › appendix S4.pdf]

MiRNAs associated with LUAD therapy.

MiRNA	Possible Mechanism	References (PMID)
miR-1	Decreases sensitivity to EGFR-TKI by changing TIME	33305905
miR-7	Reduces EGFR expression in cell lines with acquired EGFR-TKI resistance	21712475
miR-9	Reduces NFκB1 to increase sensitivity to radiotherapy ;Let-7 also, suggested to do it by decreasing Ras and IL-6	21464588
miR-10a	miR-10a increases the cisplatin resistance of lung adenocarcinoma circulating tumor cells via targeting PIK3CA in the PI3K/Akt pathway	32186774
miR-15b	miR-15b increases cisplatin resistance and metastasis by targeting PEBP4 in human lung adenocarcinoma cells	25721211
miR-15a/16	miR-15a/16 enhances radiation sensitivity of non-small cell lung cancer cells by targeting the TLR1/NF-κB signaling pathway	25442346
miR-16	We demonstrated that miR-16 directly targets the three KRAS downstream effectors MAPK3, MAP2K1, and CRAF in NSCLC, restoring the sensitivity to erlotinib (EGFR-TKI) in KRAS-mutated NSCLC both in vitro and in vivo	34948154
miR-17	miR-17-5p could inhibit the mRNA and protein levels of enhancer of zeste homolog (EZH) 1, a member of the EZH family that contributes to drug resistance, here EGFR-TKI, in several types of cancer	27633093
miR-18a	Radiosensitizing effects of miR-18a-5p on lung cancer stem-like cells via downregulating both ATM and HIF-1α	29860718
miR-20a	Cancer-associated fibroblast-derived exosomal microRNA-20a suppresses the PTEN/PI3K-AKT pathway to promote the progression and chemoresistance to cisplatin of non-small cell lung cancer	35857905
miR-20b	microRNA-20b-5p overexpression combing Pembrolizumab potentiates cancer cells to radiation therapy via repressing programmed death-ligand 1	34968160
miR-21	Transfer of extracellular vesicles containing two lncRNAs MEG3 and XIST, silences miR-21-5p and confers resistance to ALK-TKI chemotherapy.	30658414
miR-22	miR-22 enhances the radiosensitivity of small-cell lung cancer by targeting the WRNIP1	31190355
miR-23a	Inhibition of miR-23a increases the sensitivity of lung cancer stem cells to erlotinib, EGFR-TKI, through PTEN/PI3K/Akt pathway	28901474
miR-25	miR-25 reduces NSCLC cell radio-sensitivity through directly inhibiting BTG2 expression	25576360
miR-26a	Decreased MicroRNA-26a expression causes cisplatin resistance in human non-small cell lung cancer by increasing high mobility group A (HMGA)2. miR-26a decreased the expression of E2F1, diminished Akt phosphorylation, and downregulated Bcl2 expression. Cell growth was suppressed by inhibiting HMGA2-mediated E2F1-Akt pathway.	26492332
miR-26b	Exosomal microRNA-26b-5p down-regulates ATF2 to enhance radiosensitivity of lung adenocarcinoma cells	32476275
miR-27a	miR-27a increases cisplatin resistance and metastasis by targeting RKIP in human lung adenocarcinoma cells	25128483
miR-29b	Up-regulation of VANGL1 by IGF2BPs and miR-29b-3p attenuates the detrimental effect of irradiation on lung adenocarcinoma	33228740
miR-30a	Combination therapy of gefitinib and miR-30a-5p may overcome acquired drug	32552611

	resistance against EGFR-TKI through regulating through regulation of the insulin-like growth factor receptor-1 (IGF1R) and hepatocyte growth factor receptor signaling pathways which converge on the PI3K/AKT pathway in non-small cell lung cancer	
miR-30b	miR-30b-5p inhibits cancer progression and enhances cisplatin sensitivity in lung cancer through targeting LRP8	33779882
miR-31	MicroRNA-31 inhibits cisplatin-induced apoptosis in non-small cell lung cancer cells by regulating the drug transporter ABCB9	24099915
miR-32, miR-548a	MicroRNA-32 and MicroRNA-548a Promote the Drug Sensitivity of Non-Small Cell Lung Cancer Cells to Cisplatin by Targeting ROBO1 and Inhibiting the Activation of Wnt/β-Catenin Axis	33854371
miR-33a	miR-33a-5p enhances the sensitivity of lung adenocarcinoma cells to celastrol by regulating mTOR signaling	29484434
miR-34	miRNA-34 prevents cancer initiation and progression in a therapeutically-resistant (EGFR-TKI) K-ras and p53-induced mouse model of lung adenocarcinoma by decreasing antiapoptotic Met and Bcl-2	22964582
miR-96	miR-96 induces cisplatin chemoresistance in non-small cell lung cancer cells by downregulating SAMD9	26893673
miR-98	MicroRNA-98 sensitizes cisplatin-resistant human lung adenocarcinoma cells by up-regulation of HMGA2	23700794
miR-100	MiR-100 resensitizes docetaxel-resistant human lung adenocarcinoma cells (SPC-A1) to docetaxel by targeting Plk1	22120675
miR-101	miR-101-3p sensitizes lung adenocarcinoma cells to irradiation via targeting BIRC5	33732358
miR-106a	MicroRNA-106a targets autophagy and enhances sensitivity of lung cancer cells to SrcTKI inhibitors	27372519
miR-107	MiRNA-107 enhances chemosensitivity to paclitaxel by targeting antiapoptotic factor Bcl-w in non-small cell lung cancer	28979809
miR-122	MiR-122 Induces Radiosensitization in Non-Small Cell Lung Cancer Cell Line	26389880
miR-124	miR-124-3p Regulates FGF2-EGFR Pathway to Overcome Pemetrexed Resistance in Lung Adenocarcinoma Cells by Targeting MGAT5	33223850
miR-125b	miR-125b-5p upregulation by TRIM28 induces cisplatin resistance in non-small cell lung cancer through CREB1 inhibition	36476351
miR-126	The reversion of DNA methylation-induced miRNA silencing via biomimetic nanoparticles-mediated gene delivery for efficient lung adenocarcinoma therapy through targeting ADAM9 and increase of apoptosis	36171576
miR-127	EGFR-TKI	27869168
miR-129	miR-129-2 upregulation induces apoptosis and promotes NSCLC chemosensitivity to cisplatin by targeting SOX4	35146917
miR-133b	miR-133b reverses cisplatin resistance by targeting GSTP1 in cisplatin-resistant lung cancer cells	29328427
miR-134	MicroRNA-134 reverses multidrug resistance in human lung adenocarcinoma cells by targeting FOXM1	28454276
miR-135	Downregulation of MicroRNA-135 Promotes Sensitivity of Non-Small Cell Lung Cancer to Gefitinib, EGFR-TKI, by Targeting TRIM16	29295721
miR-136	Exosomal miR-136-5p Derived from Anlotinib-Resistant NSCLC Cells Confers Anlotinib, EGFR-TKI, Resistance in Non-Small Cell Lung Cancer Through Targeting PPP2R2A	34556984

miR-137	Oncogenic miR-137 contributes to cisplatin resistance via repressing CASP3 in lung adenocarcinoma	27429846
miR-138	miR-138-5p reverses gefitinib, EGFR-TKI, resistance in non-small cell lung cancer cells via negatively regulating G protein-coupled receptor 124	24582749
miR-139	MiR-139-5p Targetedly Regulates YAF2 and Mediates the AKT/P38 MAPK Signaling Pathway to Alleviate the Metastasis of Non-Small Cell Lung Cancer Cells and Their Resistance Against Cisplatin	33981163
miR-140	miR-140-3p enhances cisplatin sensitivity and attenuates stem cell-like properties through repressing Wnt/β-catenin signaling in lung adenocarcinoma cells	32765679
miR-124,miR-142	miR-124 and miR-142 enhance cisplatin sensitivity of non-small cell lung cancer cells through repressing autophagy via directly targeting SIRT1	35514612
miR-143/145	Stromal Expression of miR-143/145 Promotes Neoangiogenesis in Lung Cancer Development	26586766
miR-144	miR-144-5p Enhances the Radiosensitivity of Non-Small-Cell Lung Cancer Cells via Targeting ATF2	29850528
miR-145	MiR-145 increases the radiosensitivity of non-small cell lung cancer cells by suppression of TMOD3	34888652
miR-146b	miR-146b Reverses epithelial-mesenchymal transition via targeting PTP1B in cisplatin-resistance human lung adenocarcinoma cells	31709623
miR-147b	miR-147b-mediated TCA cycle dysfunction and pseudohypoxia initiate drug tolerance to EGFR-TKI inhibitors, osimertinib, in lung adenocarcinoma	31535082
miR-148b	miR-148b reverses cisplatin-resistance in non-small cell cancer cells via negatively regulating DNA (cytosine-5)-methyltransferase 1(DNMT1) expression	25927928
miR-150	NOTCH3 Overexpression and Posttranscriptional Regulation by miR-150 Were Associated With EGFR-TKI, gefitinib, Resistance in Lung Adenocarcinoma	30732676
miR-153	MiR-153 inhibits the resistance of lung cancer to gefitinib via modulating expression of ABCE1	31306106
miR-155	MiR-155 inhibits the sensitivity of lung cancer cells to cisplatin via negative regulation of Apaf-1 expression	22996741
miR-181a	MiR-181a reduces radiosensitivity of non-small-cell lung cancer via inhibiting PTEN	32506887
miR-181b	MiR-181b regulates cisplatin chemosensitivity and metastasis by targeting TGFβR1/Smad signaling pathway in NSCLC	26620926
miR-181c	miR-181c contributes to cisplatin resistance in non-small cell lung cancer cells by targeting Wnt inhibition factor 1	28956120
miR-182	MicroRNA-182-5p increases hedgehog signaling pathway and chemosensitivity of cisplatin-resistant lung adenocarcinoma cells via targeting GLI2	31697978
miR-185	Inhibition of miR-185-3p Confers Erlotinib Resistance Through Upregulation of PFKL/MET in Lung Cancers	34368128
miR-192	MiR-192/NKRF axis confers lung cancer cell chemoresistance to cisplatin via the NF-κB pathway	34953057
miR-195	miR-195-5p has excellent antitumor effects via inhibiting cancer cell growth, invasion, and migration, arresting the cell cycle, promoting apoptosis, and sensitizing LUAD cells to X-ray irradiation by targeting HOXA10	34925694

miR-196a	miR-196a Upregulation Contributes to Gefitinib, EGFR-TKI, Resistance through Inhibiting GLTP Expression	35163707
miR-197	miR-197-mediated CKS1B/STAT3 axis exerts tumor progression regulated by various oncogenic genes (Bcl-2, c-Myc, and cyclin D1), and PD-L1 is a putative biomarker of this axis. Furthermore, we demonstrate that a miR-197 mimic sensitizes PD-L1(high) drug-resistant cells to chemotherapy cisplatin	25597412
miR-199a	The regulation of autophagy by the miR-199a-5p/p62 axis was a potential mechanism of small cell lung cancer cisplatin resistance	35292022
miR-200b	MicroRNA-200b reverses chemoresistance of docetaxel-resistant human lung adenocarcinoma cells by targeting E2F3	22139708
miR-203	BPI-9016M, a c-Met inhibitor, suppresses tumor cell growth, migration and invasion of lung adenocarcinoma via miR203-DKK1	30613269
miR-204	MiR-204 reduces cisplatin resistance in non-small cell lung cancer through suppression of the caveolin-1/AKT/Bad pathway	30981205
miR-205	miR-205 mediates adaptive resistance to MET-TKI via ERRFI1 targeting and raised EGFR signaling	30021798
miR-206	miR-1-3p and miR-206 sensitizes HGF-induced gefitinib-resistant human lung cancer cells through inhibition of c-Met signalling and EMT. EGFR-TKI	29664235
miR-208a	Radiation-induced miR-208a increases the proliferation and radioresistance by targeting p21 in human lung cancer cells	26754670
miR-210	MiR-210 promotes a hypoxic phenotype and increases radioresistance in human lung cancer cell lines	23492775
miR-217	MicroRNA-217 functions as a tumour suppressor gene and correlates with cell resistance to cisplatin in lung cancer	25234467
miR-218	MiR-218-5p Suppresses the Killing Effect of Natural Killer Cell to Lung Adenocarcinoma by Targeting SHMT1	31124332
miR-219a	MiR-219a-5p enhances cisplatin sensitivity of human non-small cell lung cancer by targeting FGF9	30999114
miR-221	MiR-221-3p-mediated downregulation of MDM2 reverses the paclitaxel resistance of non-small cell lung cancer in vitro and in vivo	33771522
miR-223	miR-223 enhances the sensitivity of non-small cell lung cancer cells to erlotinib by targeting the insulin-like growth factor-1 receptor, EGFR-TKI	27177336
miR-224	MiR-224 promotes the chemoresistance of human lung adenocarcinoma cells to cisplatin via regulating G_1/S transition and apoptosis by targeting p21(WAF1/CIP1)	24921914
miR-296	miRNA-296-3p increases chemosensitivity to paclitaxel of lung cancer cells by targeting CX3CR1	27186308
miR-300	miR-300 decreases cellular radiosensitivity through targeting p53 and apaf1 in human lung cancer cells, DNA repair	28895780
miR-320a	MiR-320a is associated with cisplatin resistance in lung adenocarcinoma and its clinical value in non-small cell lung cancer	32731058
miR-324	miR-324-3p reverses cisplatin resistance by inducing GPX4-mediated ferroptosis in lung adenocarcinoma cell line A549	33662669
miR-335	MiR-335 decreases the chemo-radioresistance of small cell lung cancer cells by targeting PARP-1	27871924
miR-328	miRNA-328 overexpression confers cisplatin resistance in non-small cell lung cancer via targeting of PTEN	30221716
miR-337	Circ_ZNF124 promotes non-small cell lung cancer progression by abolishing	31754348

	miR-337-3p mediated downregulation of JAK2/STAT3 signaling pathway	
miR-339	miR-339-5p downregulation contributes to Taxol (paclitaxel) resistance in small-cell lung cancer by targeting α1,2-fucosyltransferase 1	28940895
miR-345	Trans-3,5,4′-trimethoxystilbene reduced gefitinib resistance in NSCLCs via suppressing MAPK/Akt/Bcl-2 pathway by upregulation of miR-345 and miR-498, EGFR-TKI	30701693
miR-365	MiR-365 enhances the radiosensitivity of non-small cell lung cancer cells through targeting CDC25A	30902389
miR-372	Upregulation of KCNQ1OT1 promotes resistance to stereotactic body radiotherapy in lung adenocarcinoma by inducing ATG5/ATG12-mediated autophagy via miR-372-3p	33082306
miR-373	Reactivation of epigenetically silenced miR-512 and miR-373 sensitizes lung cancer cells to cisplatin and restricts tumor growth	25591738
miR-376a	TRIM36 enhances lung adenocarcinoma radiosensitivity and inhibits tumorigenesis through promoting RAD51 ubiquitination and antagonizing hsa-miR-376a-5p	36058131
miR-377	The long non-coding RNA SNHG5 regulates gefitinib resistance in lung adenocarcinoma cells by targetting miR-377/CASP1 axis,EGFR-TKI	29592872
miR-378	miRNA-378 reverses chemoresistance to cisplatin in lung adenocarcinoma cells by targeting secreted clusterin	26781643
miR-379	Suppression of EIF4G2 by miR-379 potentiates the cisplatin chemosensitivity in nonsmall cell lung cancer cells	28117895
miR-381	microRNA-381 suppresses the growth and increases cisplatin sensitivity in non-small cell lung cancer cells through inhibition of nuclear factor-κB signaling	29287202
miR-383	miR-383 increases the cisplatin sensitivity of lung adenocarcinoma cells through inhibition of the RBM24-mediated NF-κB signaling pathway	34558639
miR-410	miR-410 induces both epithelial-mesenchymal transition and radioresistance through activation of the PI3K/mTOR pathway in non-small cell lung cancer	32528035
miR-425	Exosomal Transfer Of Cisplatin-Induced miR-425-3p Confers Cisplatin Resistance In NSCLC Through Activating Autophagy	31632022
miR-432	A Novel Long Non-coding RNA, MSTRG.51053.2 increases Cisplatin Resistance by Sponging the miR-432-5p in Non-small Cell Lung Cancer Cells	32158694
miR-449a	MicroRNA-449a enhances radiosensitivity in CL1-0 lung adenocarcinoma cells	23614048
miR-451	Notch-1 Confers Chemoresistance in Lung Adenocarcinoma to Taxanes through dowregulation AP-1/microRNA-451 Mediated Regulation of MDR-1, Docetaxel	27727250
miR-454	LncRNA HOXA11-AS drives cisplatin resistance of human LUAD cells via modulating miR-454-3p/Stat3	30099826
miR-483	Epigenetic silencing of miR-483-3p promotes acquired gefitinib resistance and EMT in EGFR-mutant NSCLC by targeting integrin β3,EGFR-TKI	29717264
miR-487b	miR-487b-5p increases Temozolomide Resistance of Lung Cancer Cells Through LAMP2-Medicated Autophagy	27097129
miR-488	MiR-488 inhibits proliferation and cisplatin sensitivity in non-small-cell lung cancer (NSCLC) cells by activating the eIF3a-mediated NER signaling pathway	28074905
miR-493	Epigenetic silencing of miR-493 increases the resistance to cisplatin in lung cancer by targeting tongue cancer resistance-related protein 1(TCRP1)	28859669
miR-495	miR-495 enhances the sensitivity of non-small cell lung cancer cells to platinum by modulation of copper-transporting P-type adenosine triphosphatase A	24038379

	(ATP7A)	
miR-497	miR-497 may enhance the sensitivity of non-small cell lung cancer cells to gefitinib through targeting the insulin-like growth factor-1 receptor, EGFR-TKI	30505497
miR-506	MicroRNA-506-3p reverses gefitinib resistance in non-small cell lung cancer by targeting Yes-associated protein 1,EGFR-TKI	30535506
miR-511	miR-511 induces the apoptosis of radioresistant lung adenocarcinoma cells by triggering BAX	24402374
miR-512	Hsa_circ_0005576 promotes osimertinib resistance through the miR-512-5p/IGF1R axis in lung adenocarcinoma cells, EGFR-TKI	34706132
miR-513a	miR-513a-3p sensitizes human lung adenocarcinoma cells to chemotherapy by targeting GSTP1,Cisplatin	22749944
miR-519a	Restored microRNA-519a enhances the radiosensitivity of non-small cell lung cancer via suppressing EphA2	33414521
miR-526b	miR-526b-3p inhibits lung cancer cisplatin-resistance and metastasis by inhibiting STAT3-promoted PD-L1	34321456
miR-548a	miR-548a promote the drug sensitivity of LUAD cells to cisplatin by targeting ROBO1	33854371
miR-556	Knock-down of microRNA miR-556-5p increases cisplatin-sensitivity in non-small cell lung cancer (NSCLC) via activating NLR family pyrin domain containing 3 (NLRP3)-mediated pyroptotic cell death	34488537
miR-608	miR-608 significantly contribute to the prognosis of lung adenocarcinoma treated with EGFR-TKIs	30552364
miR-613	Exo-miR-613 reversed chemoresistance to cisplatin in A549 cell	32943930
miR-630	miR-630 as novel modulators of the cisplatin response in A549 cell	20145152
miR-641	Increased expression of miR-641 contributes to erlotinib resistance in PC9 cells by targeting NF1	29493886
miR-650	MicroRNA-650 confers the docetaxel chemoresistance of lung adenocarcinoma cells via regulating Bcl-2/Bax expression	23991130
miR-660	miR-660 enhances cisplatin sensitivity via decreasing SATB2 expression in lung adenocarcinoma	37107669
miR-760	miR-760 enhances TRAIL sensitivity in non-small cell lung cancer via targeting the protein FOXA1	29665655
miR-762	miR-762 activation confers acquired resistance to gefitinib in non-small cell lung cancer	31823748
miR-764	inhibition of circ7312 decreased osimertinib resistance by promoting pyroptosis and apoptosis via the miR-764/MAPK1 axis in LUAD cells	35812182
miR-873	The inhibition of miR-873 increased gefitinib resistance of PC9 cells via the upregulation of GLI1	30126075
miR-885	miRNA-885-3p inhibits docetaxel chemoresistance in lung adenocarcinoma by downregulating Aurora A	30431113
miR-934	Cancer cell-derived exosomal circUSP7 induces CD8+ T cell dysfunction and anti-PD1 resistance by regulating the miR-934/SHP2 axis in NSCLC	34753486
miR-935	Knockdown of miR-935 increases paclitaxel sensitivity via regulation of SOX7 in A549 cells	30066948
miR-936	miR-936 targets GPR78 and improves the sensitivity of NSCLC cells to cisplatin via the Galphaq Rho GTPase pathway.	36239568
miR-1184	CircNEIL3 mediates pyroptosis to influence lung adenocarcinoma radiotherapy by upregulating PIF1 through miR-1184 inhibition	35190532

miR-1208	Silencing circPVT1 enhances radiosensitivity in non-small cell lung cancer by sponging microRNA-1208	33896835
miR-1236	Cisplatin	30805558
miR-1244	miR-1236-3p could reverse cisplatin resistance by modulation of TPT1 gene and inhibition of Pim-3 signaling pathway in A549 cells	27073334
miR-1247	miR-1247-3p targets STAT5A to inhibit lung adenocarcinoma cell migration and chemotherapy resistance	35517418
miR-1260b	miR-1260b activates Wnt signaling to regulate Taxane resistance in LUAD	33224874
miR-1262	miR-1262 expression enhanced the anticancer effects of gefitinib on LUAD cells	32343915
miR-1275	Downregulation of Linc00173 increases BCL2 mRNA stability via the miR-1275/PROCA1/ZFP36L2 axis and induces acquired cisplatin resistance of LUAD	36627670
miR-1323	Knockdown of microRNA-1323 restores sensitivity to radiation by suppression of PRKDC activity in radiation-resistant LUAD cells	25823795
miR-4513	miR-4513 significantly contribute to the prognosis of lung adenocarcinoma treated with EGFR-TKIs	30552364
miR-6077	miR-6077 promotes cisplatin/pemetrexed resistance in lung adenocarcinoma via CDKN1A/cell cycle arrest and KEAP1/ferroptosis pathways	35505963
